# Silmitasertib-induced macropinocytosis promoting DDP intracellular uptake to enhance cell apoptosis in oral squamous cell carcinoma

**DOI:** 10.1080/10717544.2021.2000677

**Published:** 2021-11-12

**Authors:** Shaojuan Song, Xin Xia, Jiajia Qi, Xiaopei Hu, Qian Chen, Jiang Liu, Ning Ji, Hang Zhao

**Affiliations:** State Key Laboratory of Oral Diseases, National Clinical Research Center for Oral Diseases, Chinese Academy of Medical Sciences Research Unit of Oral Carcinogenesis and Management, West China Hospital of Stomatology, Med-X Center for Materials, Sichuan University, Chengdu, China

**Keywords:** Oral squamous cell carcinoma (OSCC), silmitasertib, macropinocytosis, cisplatin (DDP) intracellular uptake, apoptosis

## Abstract

Cisplatin (DDP) is a first-line chemotherapeutic drug applied for the treatment of oral squamous cell carcinoma (OSCC). The anticancer activity of DDP is tightly linked to its intracellular uptake. It is unwise to increase the DDP intake by increasing the dose or shortening the dosing interval because of the severe systemic toxicity (nephrotoxicity, ototoxicity and neurotoxicity) in DDP application. The main uptake pathways of DDP include passive diffusion and active transporter transport. Therefore, finding additional uptake pathways that can improve the effective intracellular concentration of DDP is critical. Macropinocytosis, an endocytic mechanism for extracellular material absorption, contributes to the intracellular uptake of anticancer drugs. No research has been conducted to determine whether macropinocytosis can augment the intracellular uptake of DDP in OSCC cells or not. Based on that, we proved for the first time that silmitasertib (previously CX-4945) could trigger macropinocytosis, which may increase the intracellular uptake of DDP and enhance apoptosis via *in vivo* and *in vitro* experiments. We hope that our findings will inspire a new approach for the application of DDP in cancer treatment.

## Introduction

More than 90% of oral cancers are OSCC (Sritippho et al., 2015). Most patients with early OSCC can be treated with a single surgical procedure. However, palliative care based on chemotherapy has become a commonly used method for most patients with advanced OSCC, especially those who cannot undergo surgery for systemic diseases or local tumor factors (Vermorken & Specenier, 2010). Since its approval in 1979, cisplatin (DDP) has become the first-line treatment for most malignancies, including OSCC (Zhong et al., 2020; Meng et al., 2021). However, following palliative treatment, the median overall survival time and effectivity rate for patients with relapsed and metastatic OSCC were only 9–11 months and 44%, respectively (Schena et al., 2005; Olasz et al., 2010). The anticancer efficacy of DDP is positively correlated with its cellular uptake. The substantial systemic toxicity of DDP (including nephrotoxicity, ototoxicity, and neurotoxicity, among others) has led to its dose limitation and extension of dosing interval (Wensing & Ciarimboli, 2013; Harrach & Ciarimboli, 2015; Martinho et al., 2018). Therefore, it was not advisable to increase the DDP intake by increasing the dose or shortening the dosing interval. Passive diffusion and transporter uptake were the major mechanisms by which cells take in DDP (Rottenberg et al., 2021; Okada et al., 2019). Finding innovative strategies for DDP uptake in cancer cells to boost intracellular DDP concentration is a promising solution to improve the efficacy of DDP treatment.

Macropinocytosis, an endocytic mechanism for extracellular substance uptake, has been shown to contribute to the intracellular uptake of anti-cancer medications (Song et al., 2020; Zhou et al., 2020; Dai et al., 2021). Macropinocytosis has been identified as a mechanism for the endocytosis of sulfuric acid-derived nanoparticles in cervical cancer cells (Zhou et al., 2018). Furthermore, Ling et al. demonstrated that glutathione-responsive prodrug nanoparticles containing platinum candidates could infiltrate ovarian cancer cells via macropinocytosis, resulting in effective intracellular uptake and anticancer therapy (Ling et al., 2019). Macropinocytosis has also been found to aid cancer cells in absorbing nucleic acid-based drugs with cell-penetrating peptides (Niu et al., 2018). Nab-paclitaxel internalized via macropinocytosis could activate macrophages and improve the efficacy of immunotherapy in pancreatic cancer (Cullis et al., 2017). However, there was still no research on the use of macropinocytosis to improve DDP uptake in OSCC cells.

Silmitasertib has been shown to induce macropinocytosis in non-small cell lung cancer, cholangiocarcinoma, and colorectal cancer cells. It has been observed that silmitasertib-treated cholangiocarcinoma cells can produce extracellular fluid-filled vacuoles akin to macropinosomes formed by macropinocytosis (Lertsuwan et al., 2018; Recouvreux & Commisso, 2017). Silmitasertib has also been reported to induce many cytoplasmic vacuoles in colorectal cancer cells, which have been proven to be derived from macropinocytosis as observed through molecular markers (Silva-Pavez et al., 2019).

**Figure 1.**
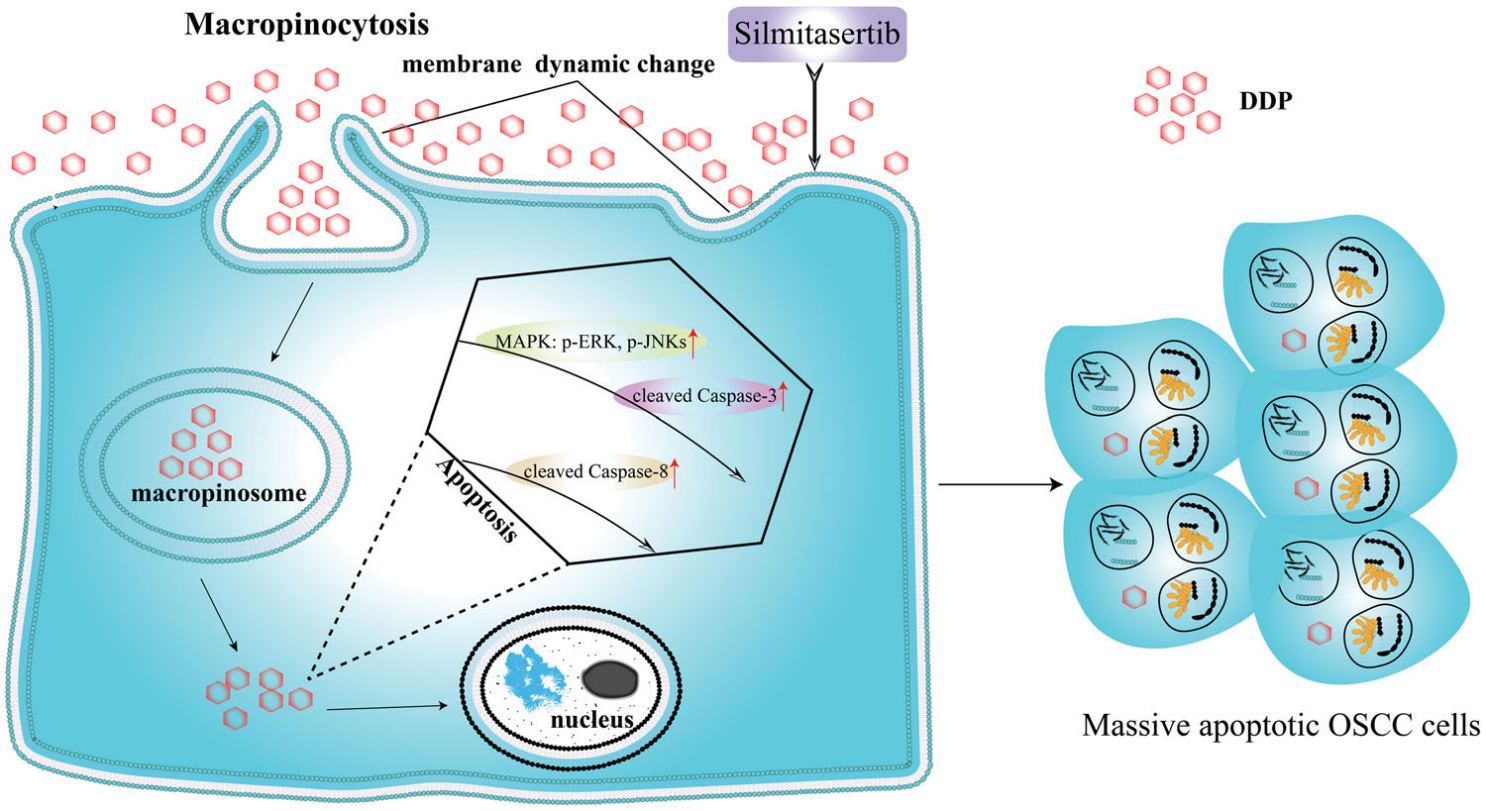
Silmitasertib-induced macropinocytosis promoting DDP intracellular uptake to enhance cell apoptosis. When silmitasertib acted on cells, the cell membrane recessed inward to form macropinosomes carrying DDP, which detached from the cell membrane and released DDP later. DDP induced strong apoptosis through the MAPK-caspase pathway in OSCC cells.

Therefore, we conducted a series of *in vitro* and *in vivo* assays to determine whether silmitasertib can stimulate macropinocytosis in OSCC cells to promote the cellular uptake of DDP. First, using phase contrast and confocal microscopy, we demonstrated that silmitasertib could induce macropinocytosis in four OSCC cell lines (Cal-27, HSC-3, HSC-4, and UM1). Second, we verified via high performance liquid phase (HPLC) that silmitasertib-induced macropinocytosis could promote the uptake of small molecule drugs DDP, 5-fluorouracil (5-FU) and isoguanosine (isoG) in OSCC cells *in vitro*. We then confirmed that silmitasertib combined with DDP might boost DPP cellular uptake, increase OSCC cell sensitivity to DDP, and enhance the apoptosis-inducing action of DDP *in vitro* by performing a CCK8 assay, flow cytometry, and western blotting (WB). Next, using a Cal-27 xenograft model established using nude BALB/c mice, we verified that silmitasertib coupled with DDP could enhance suppression of OSCC *in vivo*. Finally, we examined the inhibitory effect of silmitasertib on OSCC cells and discovered that silmitasertib could enhance apoptosis in these cells (Figure 1).

## Materials and methods

### Cell lines and reagents

OSCC cell lines (HSC-3, HSC-4) were received from the Japanese Collection of Research Bioresources (JCRB) Cell Bank and the Cal-27 and UM1 cell lines were kindly provided by Dr. J. S. Gutkind (National Institute of Dental and Craniofacial Research). The cells were routinely cultured in DMEM supplemented with 10% fetal bovine serum (Invitrogen Life Technologies) at 37 °C in 5% CO_2_ incubator. Silmitasertib, bafilomycin A1 (BAF1), Z-VAD-FMK were purchased from Selleck. Cisplatin (DDP) was purchased from Sigma-Aldrich. The Cell counting kit-8(CCK8) was purchased from Dojingdo (Kumamoto, Japan). The Annexin V-FITC/PI apoptosis double staining kit was obtained from Keygen Biotech Co. Ltd (Nanjing, China). AF488 Dextran was purchased from Thermo Fisher Scientific. Lyso-tracker Red, Mito-Tracker Red, ER-Tracker Red and Hoechst 33342 were obtained from Beyotime (Shanghai, China). The primary antibodies anti-cleaved Caspase 3, anti-cleaved Caspase 8, anti-ERK, anti-p-ERK, anti-p38, anti-JNKs, anti-p-JNKs, anti-β-actin, and anti-Ki67 were obtained from Abcam (1:1,000). Goat anti-mouse/rabbit secondary antibody was purchased from ZSGB-BIO.

### Cell viability assay by cell counting kit-8 (CCK8)

When testing the effect of silmitasertib combined with DDP treatment on the viability of Cal-27 and UM1, the cells were seeded in 96-well plates at a density of 1 × 10^4^ per well and cultured for 24 h. Then, the cells were treated with 2 μg/mL DDP, 10/20/40 μM silmitasertib or 2 μg/mL DDP + 10/20/40 μM silmitasertib for 24 and 48 h. Add 10 μL CCK8 solution to each well. And after incubating at 37 °C for 1 h, the absorbance at a wavelength of 450 nm was measured with Varioskan Flash (Thermo Scientific, USA). When testing the effect of silmitasertib on the viability of four OSCC cell lines (Cal-27, HSC-3, HSC-4, and UM1), the cells were treated with different concentrations of silmitasertib (0, 5, 10, 20, and 40 μM), and the CCK8 assay was carried out as described previously. When testing the inhibitory effect of apoptosis inhibitor Z-VAD-FMK on silmitasertib, cells were treated with 25 μM Z-VAD-FMK, 10/20/40 μM silmitasertib or 25 μM Z-VAD-FMK + 10/20/40 μM silmitasertib for 24, 48 h. Cells need to be pretreated with Z-VAD-FMK for 2 h, then silmitasertib was added, and the CCK8 assay was carried out as described previously.

### HPLC assay

The Cal-27 cells were seeded in 10 cm diameter dishes at a density of 5 × 10^5^ per dish and cultured for 24 h. And there are 8 dishes of cells in each group. Then, the cells were treated with 10 nM BAF1, 100 μM silmitasertib, 1 mg/mL DDP, 1 mg/mL DDP + 100 μM silmitasertib or 10 nM BAF1 + 1 mg/mL DDP + 100 μM silmitasertib for 4 h, respectively. Cells need to be pretreated with BAF1 for 2 h, then silmitasertib and DDP were added. Next, collect the cells into a 1.5 mL EP tube, count to ensure that the cell volume is 1 × 10^7^, wash twice with PBS, centrifuge at 2,000 rpm for 5 min, and remove the supernatant. Add 400 μL RIPA lysis buffer (50 mM tris base, 1.0 mM EDTA, 150 mM NaCl, 0.1% SDS, 1% Triton X-100, 1% sodium deoxycholate, 1% cocktail) to each tube, lyse the cells with an ultrasonic cell pulverizer (Diagenode, Belgium) at 4 °C (20 cycles, 1 min per cycle), and centrifuge at 10,000 rpm for 10 min. Take the supernatant, add 4 times the volume of methanol to precipitate the protein, centrifuge at 10,000 rpm for 10 min, and take the supernatant for HPLC (Shimadzu, Japan) detection. A Diamonsil C-18 column (4.6 mm × 250 mm, 5 μm) was used, the mobile phase was methanol-water (10:90), the flow rate was 0.5 mL/min, the detection wavelength was 254 nm, and the column temperature was 25 °C. For 5-FU and isoG, the treatment concentrations were 0.1 mg/mL and 1 mg/mL, the flow rate was 0.8 mL/min, and the detection wavelengths were 265 nm and 248 nm, respectively.

### Apoptosis assay by Annexin V-FITC/PI double staining

When testing the effect of silmitasertib combined with DDP treatment on the apoptosis of Cal-27 and UM1, the cells were seeded in 6-well plates at a density of 1 × 10^5^ per well and cultured for 24 h. Next, the cells were treated with 2 μg/mL DDP, 10 μM silmitasertib or 2 μg/mL DDP + 10 μM silmitasertib for 24 and 48 h. After removing the culture medium, cells were harvested, washed twice with PBS. Then, staining the cells with the Annexin V-FITC/PI apoptosis double staining kit according to the instructions, and detecting the apoptosis rate by flow cytometry (Beckman FC500, Brea, CA, USA). When testing the effect of silmitasertib on the apoptosis of Cal-27 and UM1, the cells were treated with different concentrations of silmitasertib (10, 20, and 40 μM), and the apoptosis assay was carried out as described previously. When testing the inhibitory effect of apoptosis inhibitor Z-VAD-FMK on silmitasertib, cells were treated with 25 μM Z-VAD-FMK, 20 μM silmitasertib or 25 μM Z-VAD-FMK +20 μM silmitasertib for 24, 48 h. Cells need to be pretreated with Z-VAD-FMK for 2 h, then silmitasertib was added, and the apoptosis assay was carried out as described previously. The data analysis was showed with Flowjo VX software. Positioning of quadrants on Annexin V/PI plots was conducted to distinguish intact (Annexin V−/PI−), necrotic (Annexin V−/PI+), early apoptotic (Annexin V+/PI−), and late apoptotic (Annexin V+/PI+) cells.

### Phase contrast microscope imaging

To observe whether silmitsertib can induce vacuoles similar to macropinocytosis in four OSCC cells (Cal-27, HSC-3, HSC-4, UM1), the cells were seeded in 6-well plates at a density of 1 × 10^5^ per well and cultured for 24 h. Then the cells were treated with silmitasertib (10, 20, and 40 μM) for 12 h, and the images were taken with a phase contrast microscope (Leica, German). When observing the inhibitory effect of the macropinocytosis inhibitor BAF1 on vacuoles induced by silmitasertib, the cells were pretreated with 10 nM BAF1 for 2 h and then treated with silmitasertib and microscope observation was carried out as described previously. Count the number of vacuoles per 100 cells from 5 pictures.

### Fluorescence confocal microscopy imaging

Cal-27 cells were seeded on a confocal dish at a density of 5 × 10^4^ per dish and cultured for 24 h. After pretreated with 10 nM BAF1 for 2 h, the cells were treated with a mixture of 20 μM silmitasertib and 0.5 mg/mL AF488 Dextran for 24 h. Then removing the culture medium, washing twice with PBS, adding hoechst 33342, Lyso-tracker Red, Mito-tracker Red, and ER-tracker Red to culture cells at 37 °C according to the instructions. Finally, removing the culture medium, washing twice with PBS, adding fresh culture medium, and the images were taken with a fluorescent confocal microscope (Olympus, Japan). Calculate the average fluorescence intensity from 5 pictures.

### Western blotting

The cell lysates were obtained through RIPA buffer (50 mM Tris base, 1.0 mM EDTA, 150 mM NaCl, 0.1% SDS, 1% Triton X-100, 1% sodium deoxycholate, 1% cocktail) and quantified by Pierce™ BCA (bicinchoninic acid) protein assay kit (Thermo Scientific™). The samples were processed to 12% sodium dodecyl sulfate polyacrylamide hydrogel electrophoresis (SDS-PAGE) at 80 V for 30 min and 120 V for 1 h and then transferred onto 0.22 μm polyvinylidene difluoride membrance (PVDF, MA, USA), using a wet transfer method at 300 mA for 75 min. The membranes were blocked with 5% skim milk in TBST for 1 h at room temperature and then incubated with primary antibodies (anti-cleaved Caspase 3, anti-cleaved Caspase 8, anti-ERK, anti-p-ERK, anti-p38, anti-JNKs, anti-p-JNKs, and anti-β-actin, Dako 1/1,000, Abcam) overnight at 4 °C. After washing with TBST for 15 min, membranes were incubated with goat anti-mouse/rabbit secondary antibody for 1 h at room temperature. Finally, the results were showed by BM Chemiluminescence Western Blotting kit (Roche).

### l-G Gels (LGBLG) encapsulating drugs to form release system

Mix 50 μL potassium hydroxide solution (1 M), 50 μL boric acid solution(1 M), 7 mg l-G (l-Guanosine) (Wuhu Nuowei Chemistry, China) and 900 μL PBS and heat (80–100 °C) until the solid powder is completely dissolved to form LGBLG solution (Yuqi Du et al., 2020). Then add 6 mg silmitasertib, 0.2 mg DDP, or 6 mg silmitasertib + 0.2 mg DDP, heat at 60 °C until the drug is completely dissolved, and cool at room temperature for 5 min until the solution turns into gel.

### Drug release study

Silmitasertib and DDP were encapsulated into LGBLG during gelation. The final concentration of silmitasertib and DDP was 6 mg/mL and 0.2 mg/mL. After preparation of the drug loaded LGBLG hydrogel (5 mL) in a vial, 500 μL of external solutions (3d H_2_O) were placed on top of the hydrogel at 37 °C for the release of the drug. 300 μL of aliquots from the upper solution were collected at different time periods. Same amount of respective buffer solution was added after every collection of aliquots to keep the volume constant. The concentration of released DDP at different time periods was detected by HPLC. And the absorbance of the solutions of simitasertib was taken by an UV − Vis spectrophotometer (PerkinElmer Lambda 750) at 265 nm. The calculation formula of the cumulative mass of released drugs *M_t_* at a time t was M_t_=C_t_×V_a_+∑(C_t−1_×V_b_) (C_t_: concentration of released drug at time t; V_a_: volume of external solution at time t = 500 μL; V_b_: volume removed from the vial every time = 300 μL). The calculation formula of the cumulative release of drug in % at time t was M_t%_=M_t_/M_total_×100 (M_total_: Total amount of drug incorporated into the hydrogel).

### Cal-27 xenograft tumor asssy

4-week-old female BALB/c athymic nude mice were purchased from the Animal Center of Sichuan University (Chengdu, China). All experiments were conducted in accordance with the guidelines outlined in the “Principles of Laboratory Animal Care” (NIH) and were approved by the local Animal Care and Use Committee. The animals had free access to sterilized water and food in a temperature-controlled room (22 ± 1 °C) with a 12 h light/dark cycle in an SPF environment. They were fed adaptively for one week in this circumstance before the experiments. The mice were injected with 100 μL Cal-27 cell suspension (2 × 10^6^ cells/mL) into the right flank. The weight of the mice and the size of the xenograft tumor were measured every other day. The calculation formula for the volume of the transplanted tumor was TV = π/6 × length×(width)^2^ (Jin et al., 2019). When the size of the xenograft tumor was close to 100 mm^3^, the mice were randomly divided into 5 groups (*n* = 6), and they were treated separately: (1) PBS, (2) 70 mg/kg LGBLG, (3) 70 mg/kg LGBLG + 2 mg/kg DDP, (4) 70 mg/kg LGBLG + 60 mg/kg silmitasertib, (5) 70 mg/kg LGBLG + 2 mg/kg DDP + 60 mg/kg silmitasertib. The drugs were peritumoral administration, once every 5 days, for four consecutive weeks, and then the experiment was ended. At the end of the experiment, all mice were sacrificed by cervical dislocation.

### Hematoxylin and eosin (H&E) staining

The collected fresh xenograft tumor tissues and main organs (heart, liver, spleen, lung and kidney) were fixed in 4% (W/V) paraformaldehyde solution for 12 h. After dehydration and paraffin embedding, the tissues were cut into 4 µm thick slices with a microtome (Leica, German) and placed on a glass slide. Then, the tissues were dewaxed by xylene for 20 min, dehydrated by gradient alcohol (100%∼5 min, 100%∼5 min, 95%∼5 min, 80%∼2 min, 70%∼2 min), dyed with hematoxylin for 30 s–1 min, and washed with running water for 15 min. After that, they were dyed with eosin for 30 s–1 min, dehydrated with gradient alcohol (80%∼2 min, 95%∼2 min, 100%∼5 min, 100%∼5 min), and transparent with xylene for 20 min. After sealing with neutral resin, the tissues were observed with a multifunctional scanner (Leica, German).

### Ki67 immunohistochemical analysis

After fixed, dehydrated, embedded, and sectioned, the tissues were deparaffinized with xylene for 20 min and dehydrated with gradient alcohol (100%∼5 min, 100%∼5 min, 95%∼5 min, 80%∼2 min, 70%∼2 min). Then, the tissues were subjected to antigen retrieval, hydrogen peroxide and serum blocking for 30 min, and incubated with the primary antibody (anti-Ki67) overnight at 4 °C, washed with PBS, then incubated with the secondary antibody for 1 h at room temperature. After washing with PBS, DAB chromogenic solution (Gene Tech, Shanghai) was used for tissue color development. Next, stain the tissues with hematoxylin for 30 s–1 min and rinse with running water for 15 min. After dehydration with gradient alcohol (80%∼2 min, 95%∼2 min, 100%∼5 min, 100%∼5 min), transparent with xylene for 20 min, and sealing with neutral resin, the tissues were observed with a multifunctional scanner (Leica, German). The arithmetical mean proportion in the five regions of Ki67-positive cells represented the proliferating cells.

### TUNEL assay

To observe apoptotic cells of tumor tissues, the TUNEL assay was performed according to the instruction of the manufacturer of the DeadEnd™ Fluorometric TUNEL System (Promega, USA). Cells showed localized green fluorescence were regarded as apoptotic cells. The arithmetical mean proportion in the five regions of apoptotic cells represented the tumor cell apoptosis. All micrographs were taken with Aperio Digital Pathology Systems.

### Statistical analysis

Experiments were performed in triplicates, or otherwise as indicated. The differences between the experimental groups and controls were assessed by single-factor analysis of variance (ANOVA) and Student's *t* test. A P-value less than .05 (*p*<.05) was regarded as a statistically significant result. The data were shown as mean value ± SD (standard deviation) except for the *in vivo* study for which the data indicate mean value ± SEM (standard error of the mean) using GraphPad Prism software (version 5.3; GraphPad Software, Inc., La Jolla, CA, USA).

## Results

### Silmitasertib could induce vacuolation in OSCC cells

**Figure 2.**
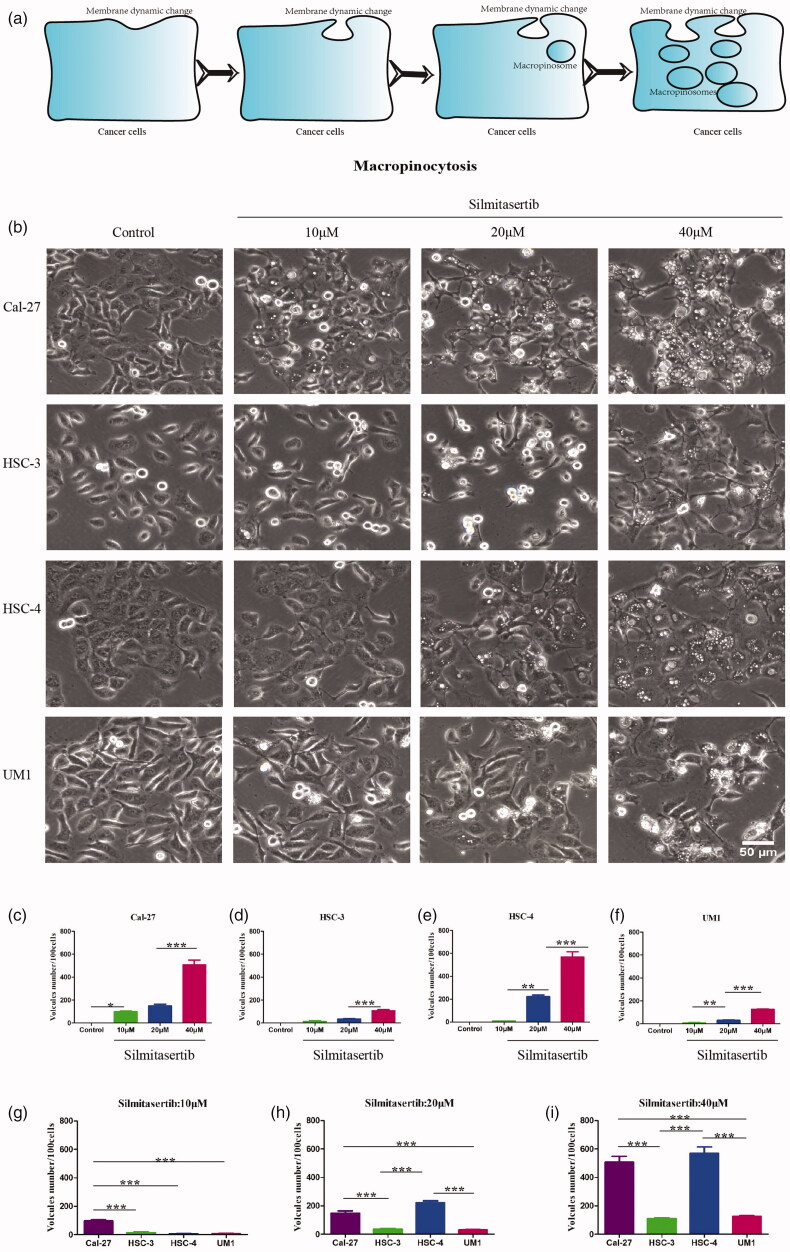
Silmitasertib can induce vacuolation in OSCC cells. **(a)** The process of macropinocytosis in cancer cells. **(b)** Cells were exposed respectively to different concentration of silmitasertib for 12 h and then their phase-contrast images were recorded by microscope. The number of vacuoles with different concentration of silmitasertib were measured in Cal-27 **(c)**, HSC-3 **(d)**, HSC-4 **(e)**, and UM1 cells **(f)**. The number of vacuoles in different cells with 10 μM **(g)**, 20 μM **(h)**, 40 μM **(i)** silmitasertib were measured. Data are shown as mean ± SD {*p<.05, **p<.01, ***p<.001} from three replicates. Scale bar: 50 μm.

Macropinocytosis was an endocytic pathway by which cells absorb extracellular fluids and substances (Mishra & Bhowmick, 2019). During this process, the cell membrane undergone dynamic changes and formed vacuoles of different sizes, typically with a diameter greater than 0.2–5 µm (Hansen & Nichols, 2009). After these vacuoles broken away from the cell membrane and entered the cell, they became macropinosomes. The main feature of macropinocytosis induced by silmitasertib in cholangiocarcinoma and rectal cancer cells was the formation of a large number of fluid-filled macropinosomes (Song et al., 2020) (Figure 2(a)).

First, to verify whether silmitasertib can induce vacuolation in OSCC cells, we treated the four types of OSCC cells(Cal-27, HSC-3, HSC-4, and UM1) with 10, 20, or 40 μM silmitasertib and observed the generation of cell vacuoles under a phase-contrast microscope after treatment for 12 h. Silmitasertib could induce the four OSCC cell lines to produce vacuoles of different numbers and sizes, and the number of vacuoles increased with an increase in the concentration of silmitasertib. Higher concentrations of silmitasertib showed more obvious differences (Figure 2(b–f)). At the same concentration (especially at 20 and 40 µM), Cal-27 and HSC-4 cells produced a large number of vacuoles, which were significantly higher than those observed in the HSC-3 and UM1 cells (Figure 2(b,g–i)). This result indicated that different cells have different sensitivities to silmitasertib. Therefore, it can be concluded that silmitasertib could cause vacuolation in OSCC cells at varying degrees depending on the concentration administered.

### The vacuoles induced by silmitasertib were deriving from macropinocytosis

**Figure 3.**
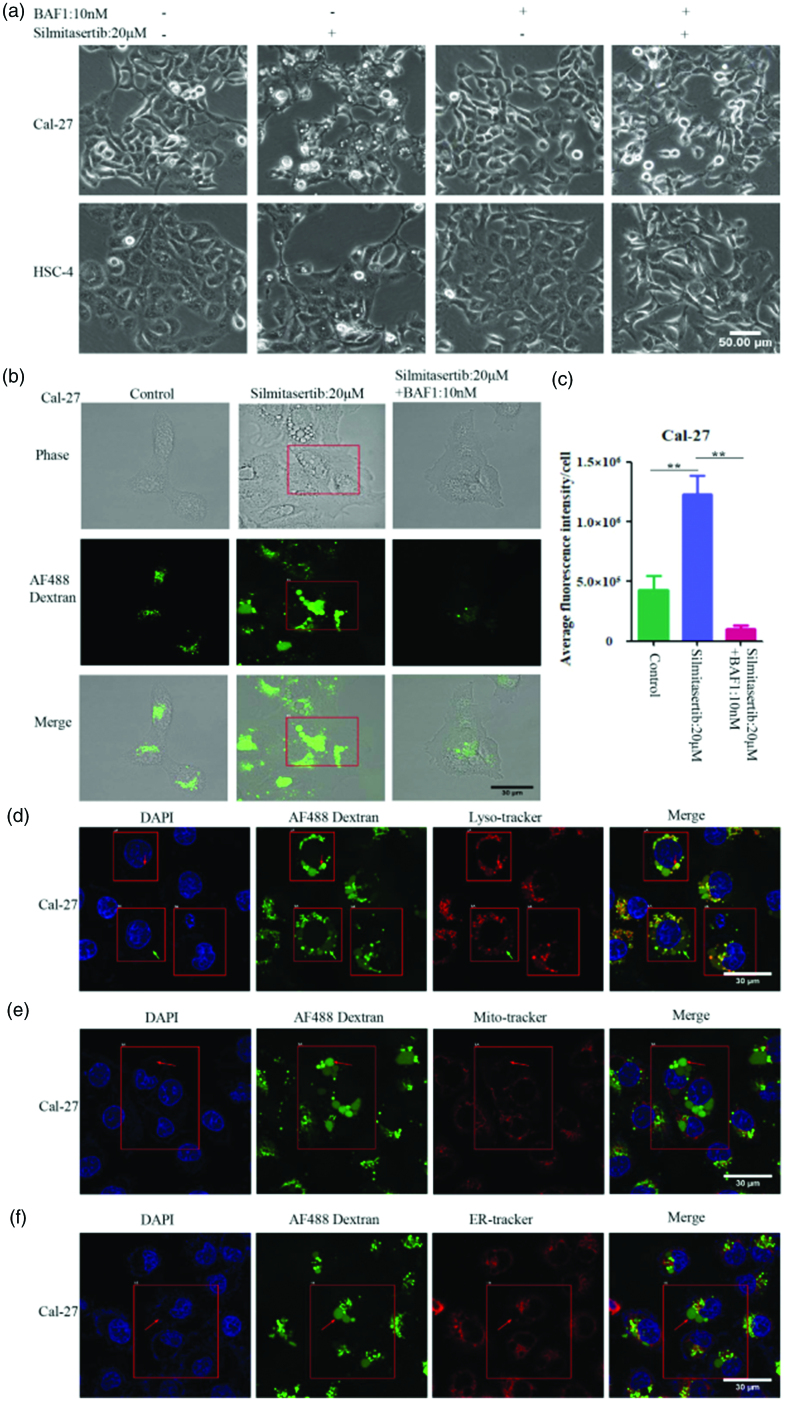
The vacuoles induced by silmitasertib were deriving from macropinocytosis. **(a)** Cells were exposed respectively to 20 μM silmitasertib or 10 nM BAF1(macropinocytosis inhibitor) or 20 μM silmitasertib + 10 nM BAF1 for 12 h and then their phase-contrast images were recorded by microscope. **(b)** Fluorescent images of macropinosomes in Cal-27 cells treated with 20 μM silmitasertib or 20 μM silmitasertib + 10 nM BAF1 were investigated by AF488 Dextran assay using microscopy. **(c)** Average fluorescence intensity in **(b)** were measured. **(d)** Fluorescent images of nucleus (DAPI), macropinosomes (AF488 Dextran) and lysosome (Lyso-Tracker) in Cal-27 cells treated with 10 μM silmitasertib for 12 h were investigated using microscopy. The red arrow indicated the macropinosomes co-localized with the lysosome, and the green arrow indicated the macropinosomes not co-localized with the lysosome. **(e–f)** Fluorescent images of nucleus (DAPI), macropinosomes (AF488 Dextran), mitochondria (Mito-Tracker) and endoplasmic reticulum (ER-Tracker) were investigated like **(d)**. The red arrow indicated the macropinosomes not co-localized with the mitochondria **(e)** or endoplasmic reticulum **(f)**. Data are shown as mean ± SD {*p<.05, **p<.01, ***p<.001} from three replicates. Scale bar: 30 μm or 50 μm.

Next, we investigated whether the vacuoles caused by silmitasertib in the above four OSCC cells were derived from macropinocytosis. We selected Cal-27 and HSC-4 cell lines that formed a large number of vacuoles as observed in the prior experiment. We first treated the cells with 10 nM BAF1 (Huang et al., 2018), a pharmacological inhibitor of macropinocytosis, 2 h before the cells were treated with 20 µM silmitasertib. We then observed the changes in the vacuoles via photography under a phase-contrast microscope 12 h after treatment with silmitasertib. After BAF1 treatment, the vacuoles induced by silmitasertib visibly disappeared, which proved that the vacuoles in OSCC cells caused by silmitasertib originated from macropinocytosis (Figure 3(a)). At the same time, we introduced the specific fluorescent marker AF488 Dextran which can label macropinosomes (Fernando et al., 2012). Cal-27 cells were selected and co-cultured with AF488 Dextran, silmitasertib, and BAF1 for 12 h. Confocal microscopy was used to obtain images of the cells, and the average intracellular fluorescence intensity was calculated. A large amount of AF488 Dextran was observed in most vacuoles, indicating the existence of macropinosomes. In the cells treated with BAF1 + silmitasertib, no vacuoles containing a large amount of AF488 Dextran were found (Figure 3(b)). The average fluorescence intensity of the silmitasertib group was significantly higher than that of the control group and the silmitasertib + BAF1 group, both *p*<.01 (Figure 3(c)).

To further confirm our results, we used different subcellular compartment dyes to determine the source of the vacuoles. After co-culturing Cal-27 cells with silmitasertib and AF488 Dextran, Lyso-tracker, Mito-tracker, and ER-tracker were used to label lysosomes, mitochondria, and the endoplasmic reticulum (ER), respectively. Some AF488 Dextran-positive vacuoles were also Lyso-tracker-positive (Figure 3(d)), consistent with the characteristics of macropinosomes in cholangiocarcinoma and rectal cancer cells (Lertsuwan et al., 2018). The vacuoles that were positive for AF488 Dextran were not co-localized with the subcellular compartments that were positive for Mito-tracker and ER-tracker, indicating that the vacuoles produced due to silmitasertib treatment were not derived from the mitochondria or the ER (Figure 3(e,f)). Therefore, we can conclude that the vacuoles in OSCC cells caused by silmitasertib were macropinosomes derived from macropinocytosis.

### Macropinocytosis induced by silmitasertib could promote DDP intracellular uptake in OSCC cells

**Figure 4.**
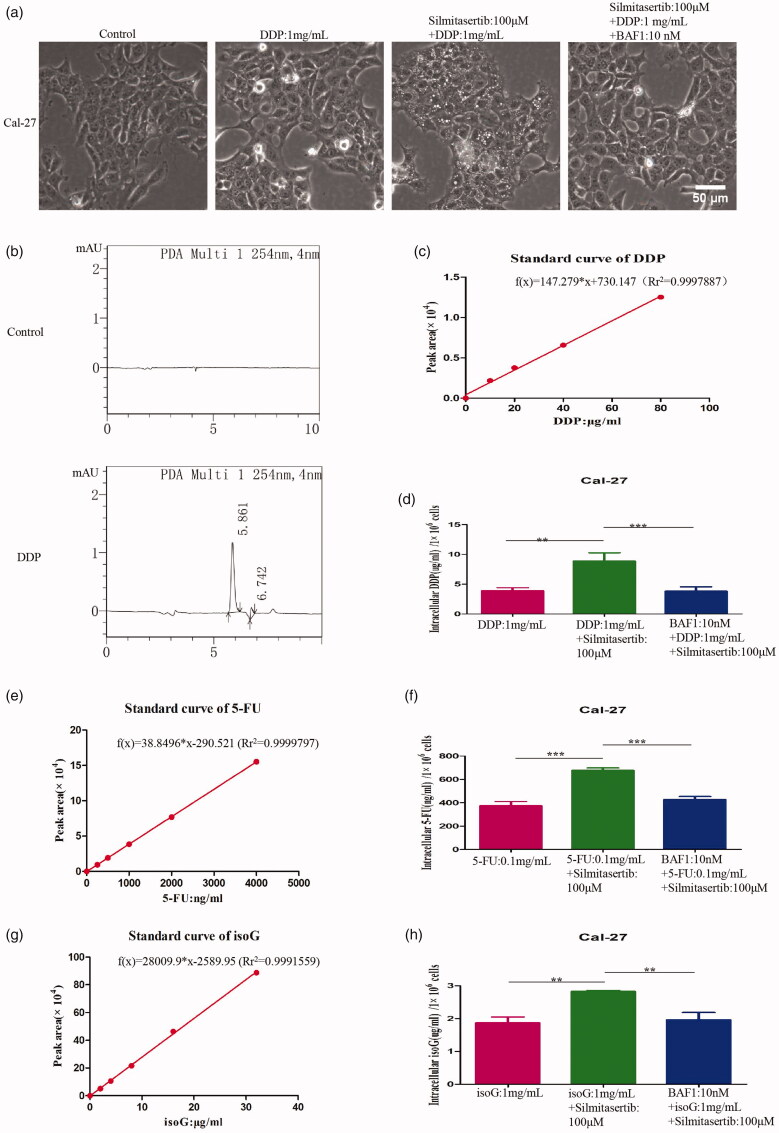
Silmitasertib could increase small molecule drugs intake in OSCC cells. **(a)** Cal-27 cells treated with 1 mg/mL DDP, 1 mg/mL DDP + 100 μM silmitasertib or 1 mg/mL DDP + 100 μM silmitasertib + 10 nM BAF1 for 4 h were observed by microscopy. **(b)** Peak figure of DDP was investigated by HPLC. **(c, e, g)** The standard curve of the drugs (DDP, 5-FU, isoG) was made with concentration as the abscissa and the peak area as the ordinate. **(d, f, h)** Intracellular drug (DDP, 5-FU, isoG) concentration in three groups were measured. Data are shown as mean ± SD {*p<.05, **p<.01, ***p<.001} from three replicates. Scale bar: 50 μm.

To further verify whether macropinocytosis induced by silmitasertib can promote the uptake of extracellular small molecule drugs in OSCC cells, DDP was selected. HPLC was used to quantify the intracellular DDP concentration. Since HPLC is not sensitive to low concentration of DDP, we choose high concentration of DDP (1 mg/mL) and silmitasertib (100 μM)) to treat Cal-27 cells for 4 h, so as to rapidly induce macropinocytosis and a large amount of DDP uptake. In addition, we also treated the cells with 10 nM BAF1 for 2 h in advance to inhibit macropinocytosis (Figure 4(a)). Through the HPLC test, the retention time of DDP was about 5.861 min (Figure 4(b)). Take DDP concentration as the abscissa and the peak area measured by HPLC as the ordinate to make a standard curve (Figure 4(c)), the function was f(x)=147.279*x + 730.147, Rr^2^ = 0.9997887. The presence of macropinocytosis significantly increased the intracellular concentration of DDP, *p*<.01; while BAF1 inhibited macropinocytosis, the intracellular concentration of DDP decreased significantly, *p*<.001 (Figure 4(d)). Compared with the DDP group, the intracellular DDP concentration of the silmitasertib + DDP group increased by 127.06% ± 7.25%, and the addition of BAF1 brought the intracellular DDP concentration back to almost the same level as the DDP group. Therefore, themacropinocytosis induced by silmitasertib could enhance the intracellular uptake of DDP.

Then, we want to know whether macropinocytosis can also increase the intracellular concentration of other drugs. We chosen two small molecule drugs, 5-fluorouracil (5-FU) and isoguanosine (isoG), and performed the same treatment (Supplementary Figures 1(a,b)). The retention time of 5-FU and isoG were 6.349 min and 10.463 min, respectively (Supplementary Figures 1(c,d)). And, the standard curve functions of 5-FU and isoG were f(x) = 38.8496*x–290.521 (Rr^2^ = 0.9999797) and f(x) = 28009.9*x–2589.95 (Rr^2^ = 0.9991559) (Figure 4(e,g)). Macropinocytosis could significantly increase the intracellular concentration of 5-FU and isoG, *p*<.001 and *p*<.01, respectively (Figure 4(f,h)); while BAF1 could reverse this change, *p*<.001 and *p*<.01, respectively (Figure 4(f,h)). Compared with the 5-FU and isoG group, the intracellular 5-FU and isoG concentration of the silmitasertib + 5-FU group and silmitasertib + isoG group increased by 87.18%±39.70% and 54.08%±21.97%, respectively. Therefore, we could conclude that macropinocytosis induced by silmitasertib can promote the intracellular absorption of small molecule drugs (DDP, 5-FU, isoG) in Cal-27 cells, and it may be universal.

### Combination of silmitasertib with DDP could promote OSCC cell apoptosis

**Figure 5.**
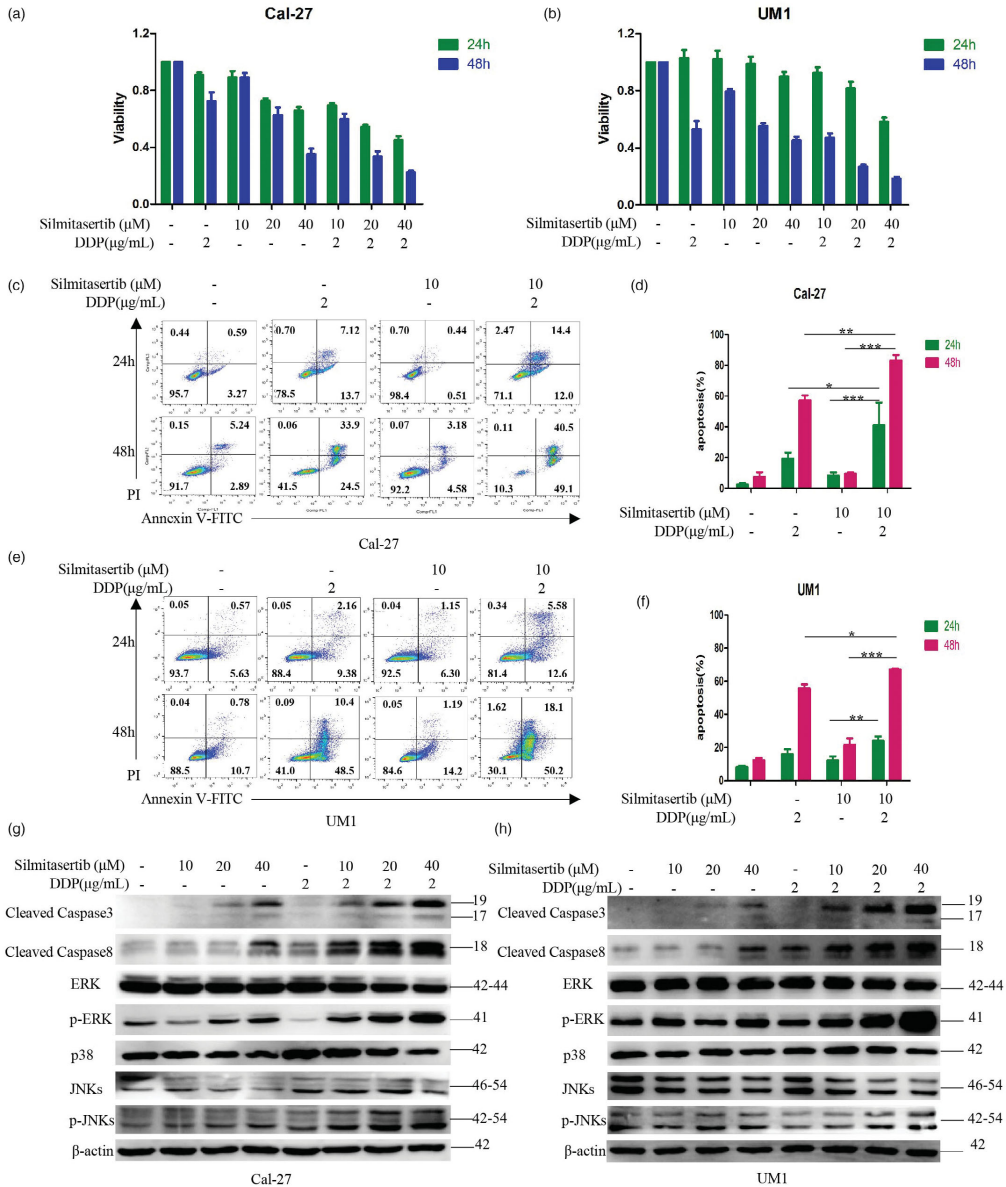
Combination of silmitasertib with DDP could promote OSCC cell apoptosis. **(a, b)** Cell viability of Cal-27 and UM1 cells treated with 2 μg/mL DDP, 10/20/40 μM silmitasertib or 2 μg/mL DDP + 10/20/40 μM silmitasertib for 24 and 48 h were measured by CCK8 assay. **(c, e)** Apoptosis in Cal-27 and UM1 cells treated with 2 μg/mL DDP, 10 μM silmitasertib or 2 μg/mL DDP + 10 μM silmitasertib for 24 and 48 h were conducted using Annexin V-FITC and propidium iodide (PI) double staining. **(d, f)** Rates of cell apoptosis in **(c, e)** were measured. **(g, h)** Cal-27 and UM1 cells were treated with 2 μg/mL DDP, 10/20/40 μM silmitasertib or 2 μg/mL DDP + 10/20/40 μM silmitasertib for 12 h, and the protein level of cleaved Caspase 3, cleaved Caspase 8, ERK, p-ERK, p38, JNKs, p-JNKs were assessed by WB. Data are shown as mean ± SD {* *p*<.05, ** *p*<.01, *** *p*<.001} from three replicates.

Next, we want to see if the increase in intracellular DDP caused by silmitasertib-induced macropinocytosis could improve the inhibition of OSCC cells. First, administered 2 µg/mL DDP combined with 10, 20, 40 μM silmitasertib to Cal-27 and UM1 cells and measured their viability after 24 and 48 h. The new bliss independence model calculation formula (Zhao et al., 2014) was used to calculate the combined inhibition rate of the two drugs. Two drugs had a synergistic effect when the theoretical inhibition rate was less than the actual inhibition rate. The two drugs had an antagonistic effect when the theoretical inhibition rate was greater than the actual inhibition rate. When the theoretical and actual inhibition rates were equal, there was neither synergy nor antagonism between the two drugs. Compared with the single-drug group, the cell viability of the combined group was significantly decreased (Figure 5(a,b), Supplementary Figures 2(a,b)). At 24 and 48 h, the combination of DDP and silmitasertib showed different degrees of synergy in Cal-27 and UM1 cells (Table 1). These results led us to believe that macropinocytosis contributes to the potent combined effect of silmitasertib and DDP in OSCC cells *in vitro*.

**Table 1.** Inhibition rates of cell viability.

Cell lines	Time(h)		Silmitasertib (10 μM) +DDP(2 μg/mL)	Silmitasertib (20 μM)+DDP(2μg/mL)	Silmitasertib (40 μM)+DDP(2 μg/mL)
Cal-27	24	actual value	30.59 ± 1.55	45.74 ± 1.20	55.15 ± 2.51
		theoretical value	19.17 ± 4.43	34.22 ± 2.44	40.50 ± 2.78
	48	actual value	40.25 ± 3.19	66.59 ± 3.11	77.70 ± 1.10
		theoretical value	35.49 ± 6.52	54.62 ± 6.49	74.51 ± 4.29
UM1	24	actual value	7.57 ± 3.43	18.28 ± 3.84	41.73 ± 2.47
		theoretical value	−5.14 ± 5.94	−1.39 ± 4.17	7.64 ± 6.68
	48	actual value	57.29 ± 2.81	73.14 ± 1.24	81.74 ± 1.15
		theoretical value	55.67 ± 4.27	70.73 ± 3.66	75.97 ± 3.27

Then, we treated Cal-27 and UM1 cells with 2 µg/mL DDP combined with 10 μM silmitasertib for 24 and 48 h. The cells were then double-stained with Annexin V-FITC and propidium iodide (FITC/PI) and were observed via flow cytometry to detect the proportion of apoptotic cells. The new bliss independence model calculation formula was then used to calculate the combined apoptosis rate of the two drugs. After a combined treatment of silmitasertib and DDP in Cal-27 cells, the apoptosis rate increased significantly, showing an obvious synergistic effect at 24 and 48 h, *p*<.05 and *p*<.01 (Figure 5(c,d)). The theoretical cooperative apoptosis rates in Cal-27 cells at 24 and 48 h were 25.78%±5.83% and 61.32%±4.50%, respectively, and the actual cooperative apoptosis rates were 40.84%±10.19% and 83.23%±4.96%, respectively. For UM1 cells, the combined treatment with silmitasertib and DDP increased the apoptosis rate at 48 h (*p*<.05), but only showed a slight synergistic effect (Figure 5(e,f)). The theoretical cooperative apoptosis rate of UM1 cells at 48 h was 65.13%±3.83%, and the actual cooperative apoptosis rate was 66.97%±0.96%. From the previous experimental results, it can be seen that after treatment with the same concentration of silmitasertib, Cal-27 cells had a significantly increased macropinocytosis activity than UM1 cells **(**both *p*<.001; Figure 2(g–i)). Therefore, we speculated that macropinocytosis could carry part of DDP into cells, causing Cal-27 cells to take up more DDP than UM1 cells, leading to a stronger pro-apoptotic effect in Cal-27 cells. These results indicated that silmitasertib combined with DDP can promote the sensitivity of OSCC cells to DDP and enhance cell apoptosis, where an increased macropinocytosis activity resulted in an increase in cell apoptosis.

Finally, we treated Cal-27 and UM1 cells with 2 µg/mL DDP combined with 10, 20, and 40 µM silmitasertib for 12 h and then measured the expression levels of three parallel MAPK signaling pathway-related proteins (p38, ERK, and JNKs) associated with cell proliferation and survival, and two other proteins (cleaved Caspase-3 and cleaved Caspase-8) which are closely related to apoptosis. Compared with the control group, the protein levels of cleaved Caspase-3, cleaved Caspase-8, p-ERK, and p-JNKs were higher after treatment with different concentrations of silmitasertib in both Cal-27 and UM1 cells. The protein levels of ERK, p38, and JNKs did not change significantly (Figure 5(g,h)). This indicated that silmitasertib could induce OSCC cell apoptosis, which was related to the MAPK-Caspase signaling pathways. Under the combined effect of silmitasertib and DDP, compared with the single-drug-treated group, the levels of cleaved Caspase-3, cleaved Caspase-8, p-ERK, and p-JNKs were higher in the combined drug group and increased with an increase in the concentration of silmitasertib in both Cal-27 and UM1 cells (Figure 5(g,h)). Compared with the single-drug group, the protein levels of ERK, p38, and JNKs in the combination group did not change significantly (Figure 5(g,h)). These results verified that the combination of silmitasertib and DDP increased apoptosis in Cal-27 and UM1 cells from the protein level.

Therefore, we could conclude that silmitasertib combined with DDP could enhance the sensitivity of some OSCC cell lines to DDP and enhance cell apoptosis, which may be related to silmitasertib-induced macropinocytosis, promoting the cell uptake of DDP.

### Combination of silmitasertib with DDP can enhance OSCC tumor suppression *in vivo*

In order to verify whether silmitasertib combined with DDP could enhance the sensitivity of tumor cells to DDP and achieve stronger tumor suppression *in vivo*, we established a Cal-27 xenograft model using female BALB/c nude mice. When the transplanted tumor reached a volume of 100 mm^3^, we administered 2 mg/kg of DDP and 60 mg/kg of silmitasertib via the peritumoral route. We used a supramolecular nucleoside hydrogel with good biocompatibility, l-G gels (LGBLG) (Yuqi Du et al., 2020), to reduce the frequency of DDP administration. Before administration, silmitasertib and DDP were encapsulated in the gel, which was applied via peritumoral administration to achieve sustained drug release. The release study of the drugs loaded LGBLG was investigated at different time periods (Supplementary Figures 3(a–c)). Almost 99% of silmitasertib was released, whereas only 87% of DDP was released after 5 days (Supplementary Figure S3(c)).

**Figure 6.**
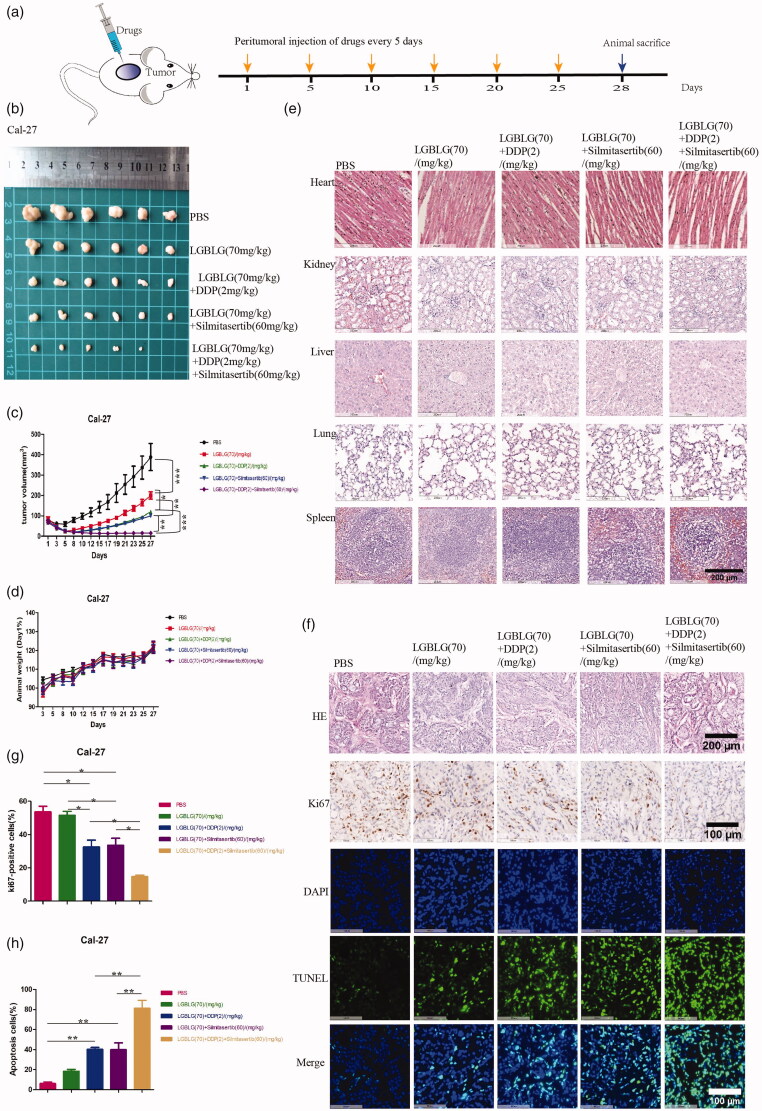
Combination of silmitasertib with DDP could enhance OSCC tumor suppression *in vivo*. **(a)** The drugs were administered once every five days for four weeks, after which the mice were sacrificed and the tumor tissues were collected. **(b)** The image of Cal-27 xenografts at the end of the experiment. **(c)** Average tumor growth curves in control and treatment groups receiving the indicated treatment. **(d)** Average animal body weight curves in control and treatment groups. **(e)** Hematoxylin and eosin (H&E) staining images were observed in main organs (heart, kidney, liver, lung and spleen) at the end of the experiment. **(f)** The histological, cell proliferation, and cell apoptosis of Cal-27 xenografts tissues in different groups were performed by H&E staining, immunohistochemical method (Ki67) and the TUNEL assay, respectively. **(g, h)** Rates of Ki67 positive cells and apoptosis cells in Cal-27 xenograft tissues of different groups were measured. Data are shown as mean ± SEM {*p<.05, **p<.01, ***p<.001}. Scale bar: 100 μm, 200 μm.

We administered the drug once every five days for four weeks, after which we sacrificed the mice and collected the tumor tissues (Figure 6(a)). With the prolongation of the administration time, the tumor suppression effect of the combination group was enhanced compared to that of the single-agent group (Figure 6(c)). At the end of the experiment, the tumor volume of the silmitasertib + DDP group was statistically smaller than that of the silmitasertib- and DDP-treated groups (*p*<.01 and *p*<.001, respectively). At the same time, the tumor volume in the combination group was visually smaller than that in the single-agent group, and the tumor in one mouse in the combined group disappeared completely (Figure 6(b)). The combined inhibition rate of silmitasertib combined with DDP was calculated using the new bliss independence model calculation formula. The theoretical combined tumor inhibition rate of silmitasertib and DDP was 88.71%±6.73%, while the actual combined inhibition rate was 95.46%±3.67%. This result indicated that the combination of silmitasertib and DDP also had a synergistic effect *in vivo*. During the experiment, the bodyweight of the mice in each group showed a steady growth trend (Figure 6(d)), indicating that silmitasertib combined with DDP could achieve a stronger tumor suppression effect than monotherapy *in vivo*, with a low systemic toxicity. To further investigate the systemic toxicity of the combination of silmitasertib and DDP, we performed hematoxylin and eosin (H&E) staining of the internal organs of the mice to observe histological changes. The results showed that the combination of silmitasertib and DDP has low systemic toxicity (Figure 6(e)).

Next, we performed H&E staining, Ki67 immunohistochemical staining, and the TUNEL assay on the collected tumor tissues to determine the proliferation and apoptosis in the cells within the tumor. The tumor proliferation positively related to ki67 of the combined silmitasertib and DDP group was significantly lower than that of the silmitasertib- and DDP-treated groups (*p*<.05) (Figure 6(f,g)). In contrast, the tumor apoptosis rate positively related to TUNEL of the combination group was significantly higher than that of the two single-agent-treated groups (*p*<.01) (Figure 6(f,h)). Therefore, we could conclude that silmitasertib combined with DDP could exhibit a stronger tumor suppression and apoptosis induction effect than single-drug therapy *in vivo*, and the drugs had low systemic toxicity.

## Discussion

First, we discovered that four OSCC cell lines (Cal-27, HSC-3, HSC-4, and UM1) showed varying degrees of macropinocytosis activity after treatment with silmitasertib (Figure 2(a–i), Figure 3(a–f)). Cal-27 and HSC-4 cells had higher macropinocytosis activity than HSC-3 and UM1 cells after treatment with the same quantity of silmitasertib, suggesting that different types of cells have different sensitivities to silmitasertib. Cholangiocarcinoma cell lines (HuCCA-1, CCLP-1, and KKU-M213), breast cancer cell lines (MDA-MB-231 and T47D), and a prostate cancer cell line (DU145) treated with silmitasertib all demonstrated substantial macropinocytosis activity, according to the study by Lertsuwan et al. On the other hand, the prostate cancer cell line PC3 and breast cancer cell line MCF-7 did not produce or only formed a few vacuoles under identical experimental conditions (Lertsuwan et al., 2018). This indicated that silmitasertib did not consistently induce macropinocytosis in all tumor cells, and drug sensitivity may be related to the cell type. Macropinocytosis has been linked to several RAS genes (e.g. K-RAS and H-RAS) (Belaid & Filippakis, 2021; Kay, 2021), small GTPases (e.g. Cdc42, Rac, Rab, and RhoA) (Garrett et al., 2000; Somsel Rodman & Wandinger-Ness, 2000; Pertz et al., 2006; Valsalakumari et al., 2021; Buckley et al., 2020), and phosphoinositides (Di Paolo & De Camilli, 2006; Walpole & Grinstein, 2020). Changes in the activity of these genes and related factors may play a role in the differences in macropinocytosis activity between various cell types.

Second, macropinocytosis has been identified as one of the routes by which cells take up drug molecules (Niu et al., 2018; Gao et al., 2017; Wang et al., 2013). We aimed to determine whether silmitasertib-induced macropinocytosis promotes the uptake of extracellular drugs in OSCC cells. Three small-molecule drugs, DDP, 5-FU and isoG, were selected to confirm our conjecture via HPLC. We observed that macropinocytosis induced by silmitasertib could indeed promote the uptake of extracellular small molecule drugs in OSCC cells (Figure 4(a–h)). Existing studies on the uptake of drugs via macropinocytosis mainly focused on drug nanoparticles and drug conjugates, including albumin-bound paclitaxel (Cullis et al., 2017)and self-assembled nanoparticles loaded with platinum prodrugs (Ling et al., 2019). In these studies, the active drug was coated or linked to a molecule that can cause tumor cells to undergo macropinocytosis. The active drug relies on macropinocytosis caused by this chemical structure to enter the cell. However, our findings suggest that using macropinocytosis generated by silmitasertib to directly increase the uptake of extracellular drugs in cancer cells could be a simpler solution. This avoids the time-consuming process of synthesizing drug nanoparticles or drug conjugates.

Next, we wanted to determine whether silmitasertib-induced macropinocytosis could increase DDP accumulation in OSCC cells. As one of the first-line chemotherapeutics for OSCC, the severe systemic toxicity of DDP has led to a dose limitation and extension of dosing interval (Wensing & Ciarimboli, 2013; Harrach & Ciarimboli, 2015; Martinho et al., 2018). Furthermore, the anticancer efficacy of DDP was linked to its cell uptake (Rottenberg et al., 2021; Okada et al., 2019). We initially tested whether silmitasertib coupled with DDP could improve the sensitivity of OSCC cells to DDP *in vitro*. The sensitivity of cells to DDP and apoptosis were greatly increased when both silmitasertib and DDP were administered in combination (Figure 5(a–h)). In addition, apoptosis of Cal-27 cells increased at a substantially faster rate than UM1 cells. Consistently, Cal-27 cells produced greater macropinocytosis activity than UM1 cells upon treatment with the same concentration of silmitasertib (Figure 2(a–i), Figure 3(a–f)). Therefore, we believed that what was related to the high rate of macropinocytosis in Cal-27 cells, which delivered a portion of the extracellular DDP into the cells, boosting intracellular DDP accumulation, DDP efficacy, and promoting cell apoptosis. Although the combination of the two medications accelerated the apoptosis of UM1 cells, the combined effect in UM1 cells was not as visible as in Cal-27 cells due to the weaken macropinocytosis. In addition to DDP, silmitasertib can enhance the sensitivity of tumor cells to other anticancer drugs, including paclitaxel (Jung et al., 2019) and platinum prodrugs (Chen et al., 2020). However, the role of macropinocytosis in drug uptake has not yet been determined. Macropinocytosis induced by silmitasertib in various tumor cells may promote the cellular uptake of multiple anticancer drugs. However, the level of macropinocytosis activity depending on different parameters warrants further investigation.

*In vivo*, we confirmed that administration of a combination of silmitasertib and DDP resulted in greater tumor suppression and cell apoptosis than monotherapy (Figure 6(a–h)), which was in line with our *in vitro* findings. The use of silmitasertib with DDP to increase cancer cell sensitivity to DDP has been investigated in a number of malignancies, including non-small cell lung cancer, cholangiocarcinoma, cervical cancer, and ovarian cancer (Zakharia et al., 2019; Rabalski et al., 2016; Siddiqui-Jain et al., 2012; Kildey et al., 2021). These studies focused on drug resistance in cancers but seldom paid attention to promoting DDP uptake through macropinocytosis induced by silmitasertib. DDP drug resistance was linked to a reduction in cellular uptake and an increase in efflux (Harrach & Ciarimboli, 2015; Martinho et al., 2018).Perhaps macropinocytosis could be an auxiliary means of reducing cell resistance to DDP. Overall, our findings suggested a simpler solution to allow the active drug DDP to enter cells along with the macropinocytosis induced by silmitasertib, thereby expanding the mechanisms by which cells can take up DDP, resulting in a more effective tumor suppressor effect. However, more data are needed to show that silmitasertib-induced macropinocytosis could promote the uptake of DDP in cancer cells.

Finally, we examined the toxicity of silmitasertib on OSCC cells. Through the CCK8 assay, we found that the viability of the four OSCC cell lines used in this study (HSC-3, HSC-4, Cal-27, UM1) decreased depending on silmitasertib concentration (Supplementary Figure 4(a)). Apoptosis induced by silmitasertib also increased in a concentration-dependent manner (Supplementary Figures 4(b,c)), and the addition of the caspase-dependent apoptosis inhibitor Z-VAD-FMK (Van Noorden, 2001) reversed apoptosis in OSCC cells as confirmed via FITC/PI double labeling (Supplementary Figures 4(d–i)). These results indicated that silmitasertib could induce caspase-dependent apoptosis in OSCC cells. When combined with other chemotherapeutic drugs, the toxicity of silmitasertib may be conducive to effectively suppressing tumor growth. In addition, the systemic toxicity of DDP mainly stemmed from the fact that DDP did not selectively reach tumor sites and key organs throughout the body via blood circulation and did not have tumor-targeting properties. However, as a selective ATP-competitive CK2(casein kinase 2) inhibitor, silmitasertib could target CK2 (So et al., 2015; Tang et al., 2020; Afzal et al., 2020). Many cancers showed abnormally elevated CK2 levels (Lertsuwan et al., 2018; Silva-Pavez et al., 2019; D'Amore et al., 2020), and the combined application of silmitasertib and DDP may better target cancer cells to induce macropinocytosis, thereby promoting the intracellular uptake of DDP at the tumor site. Thus, the systemic toxicity of DDP may be reduced while its efficacy is improved.

## Conclusion

Here, we investigated for the first time whether the macropinocytosis induced by silmitasertib could be exploited as an additional method for OSCC cells to take up DDP. First, we confirmed *in vitro* that silmitasertib could trigger macropinocytosis in OSCC cells using macropinocytosis inhibitors and the corresponding molecular markers. Second, we verified via HPLC that the macropinocytosis induced by silmitasertib could promote the absorption of small molecule drugs DDP, 5-FU, and isoG in OSCC cells *in vitro*. Next, the CCK8 test, FITC/PI apoptosis double staining assay, and WB were utilized to confirm that silmitasertib coupled with DDP could augment OSCC cell sensitivity to DDP and increase cell apoptosis *in vitro*. We also verified that silmitasertib combined with DDP could achieve *in vivo* tumor suppression with high efficiency. Finally, we conducted a preliminary exploration of OSCC growth inhibition by silmitasertib and discovered that silmitasertib could enhance apoptosis in OSCC cells. We anticipate that our findings will inspire a new approach for novel applications of DDP in OSCC treatment, namely, that silmitasertib-induced macropinocytosis-assisted DDP absorption could lead to enhanced OSCC suppression.

## Supplementary Material

Supplemental Material
