# Supplementary material for: Silmitasertib-induced macropinocytosis promoting DDP intracellular uptake to enhance cell apoptosis in oral squamous cell carcinoma
Source: Drug Deliv. 2021 Nov 12;28(1):2480–94. doi: 10.1080/10717544.2021.2000677 (PMC8592591; doi:10.1080/10717544.2021.2000677)
Supplement: Supplemental Material [file IDRD_A_2000677_SM9018.docx]

**Supporting information for:**

**Silmitasertib-induced macropinocytosis promoting** **DDP** **i****ntracellular uptake to enhance cell** **apoptosis in oral squamous cell carcinoma**

**Shaojuan Song****^+^, Xin Xia^+^, Jiajia Qi,** **Xiaopei Hu,** **Qian chen, Jiang Liu, Ning Ji***** and Hang Zhao***

State Key Laboratory of Oral Diseases, National Clinical Research Center for Oral Diseases, Chinese Academy of Medical Sciences Research Unit of Oral Carcinogenesis and Management, West China Hospital of Stomatology, Med-X Center for Materials, Sichuan University, Chengdu, China

***Correspondence:**

Ning Ji

Email: jining_1023@126.com

Hang Zhao

Email: [zhaohangahy@scu.edu.cn](mailto:zhaohangahy@scu.edu.cn)

^+^These authors contributed equally to this work.

**Key words: oral squamous cell carcinoma (OSCC), silmitasertib, macropinocytosis, cisplatin (DDP) intracellular uptake,** **apoptosis**

**Supplemental figures**

**
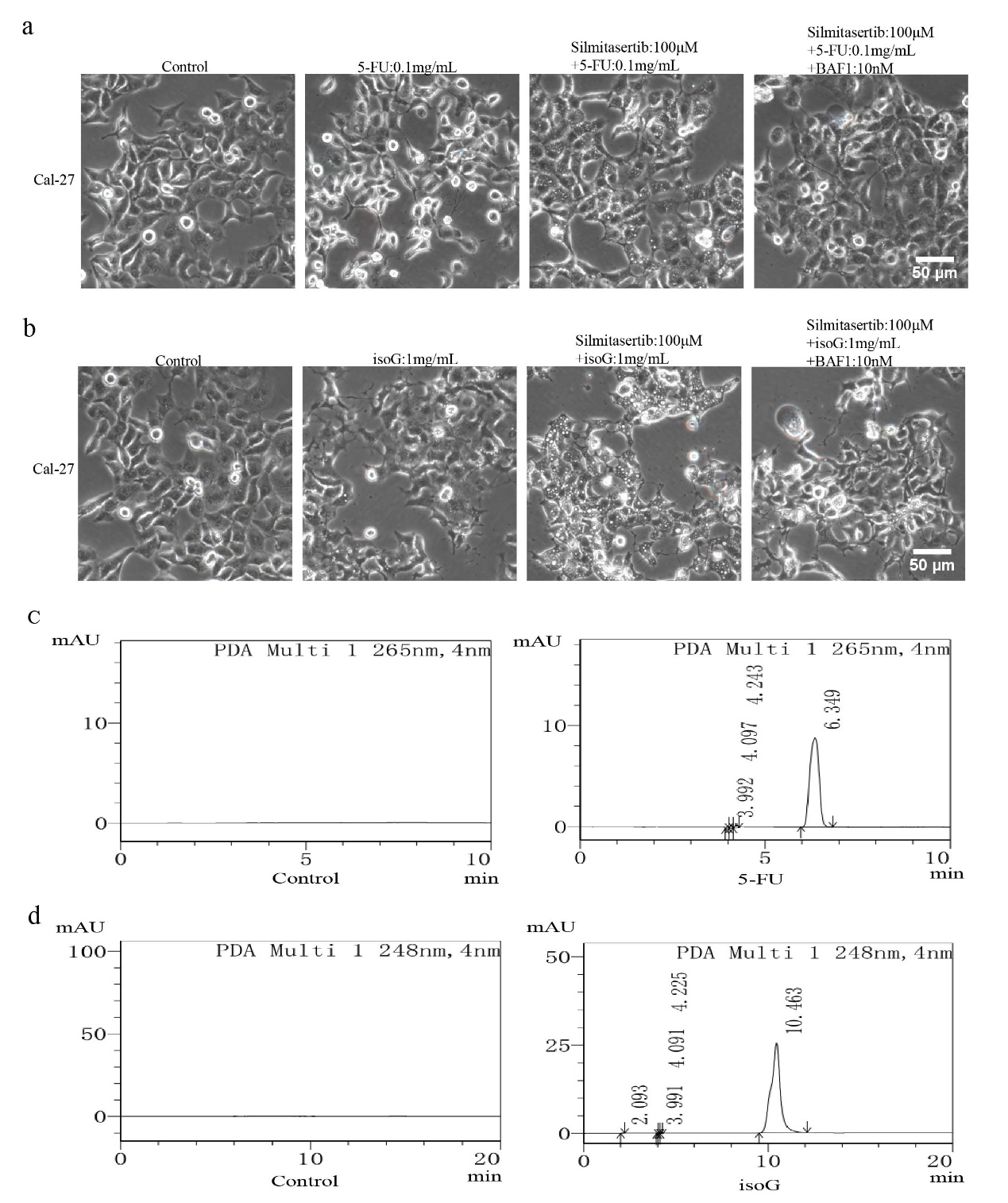
**

**Fig.S1** Macropinocytosis could increase intracellular uptake of 5-FU and isoG in Cal-27 cells. **(a, b)** Cal-27 cells treated with 0.1 mg/mL 5-FU(or 1 mg/mL isoG), 0.1 mg/mL 5-FU(or 1 mg/mL isoG)+100 μM silmitasertib or 0.1 mg/mL 5-FU(or 1 mg/mL isoG) +100 μM silmitasertib+10 nM BAF1 for 4 h was observed by microscopy. **(c, d)** Peak figure of 5-FU and isoG were investigated by HPLC.


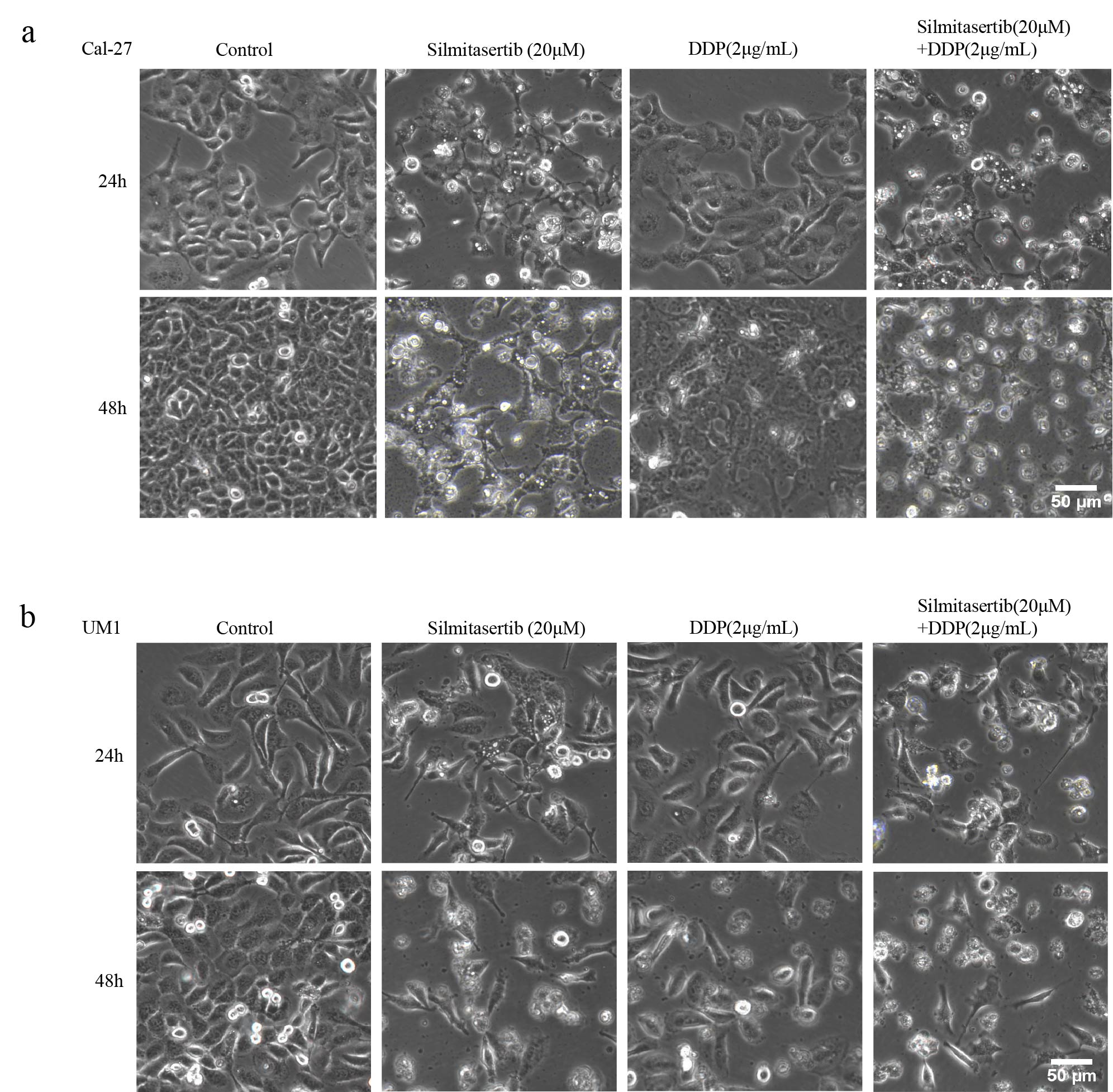


**Fig.S2** Combination of silmitasertib with DDP can promote OSCC cell inhibition. Cal-27 **(a)** and UM1 **(b)** Cells were exposed respectively to 20 μM silmitasertib or 2 μg/mL DDP or 20 μM silmitasertib+2 μg/mL DDP for 24 and 48 h and then their phase-contrast images were recorded by microscope.


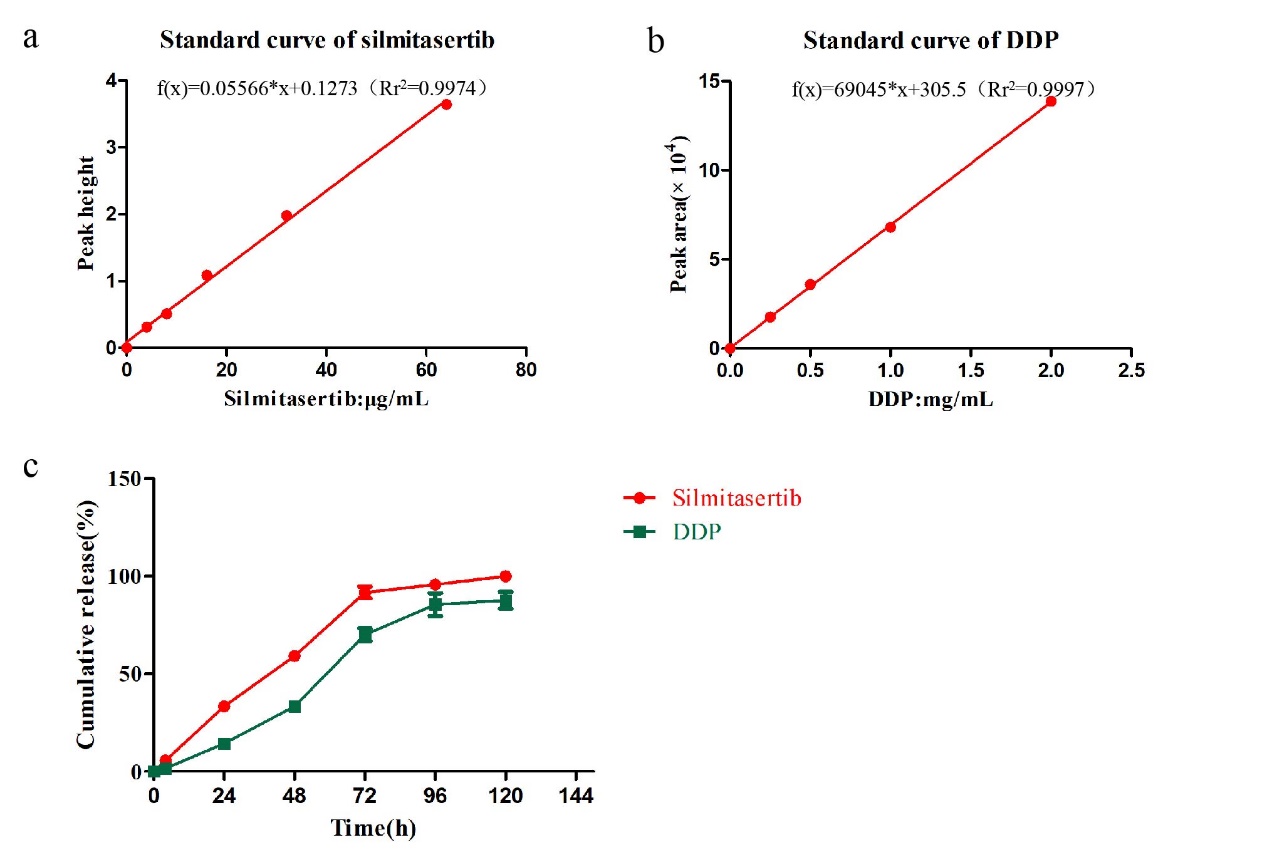


**Fig.S3** Drug release profile from LGBLG hydrogel at different time periods. **(a, b)** The standard curve of the drugs (silmitasertb and DDP) was made with concentration as the abscissa and the peak height/area as the ordinate. **(c)** The cumulative release of silmitasertib and DDP at different time periods.

**
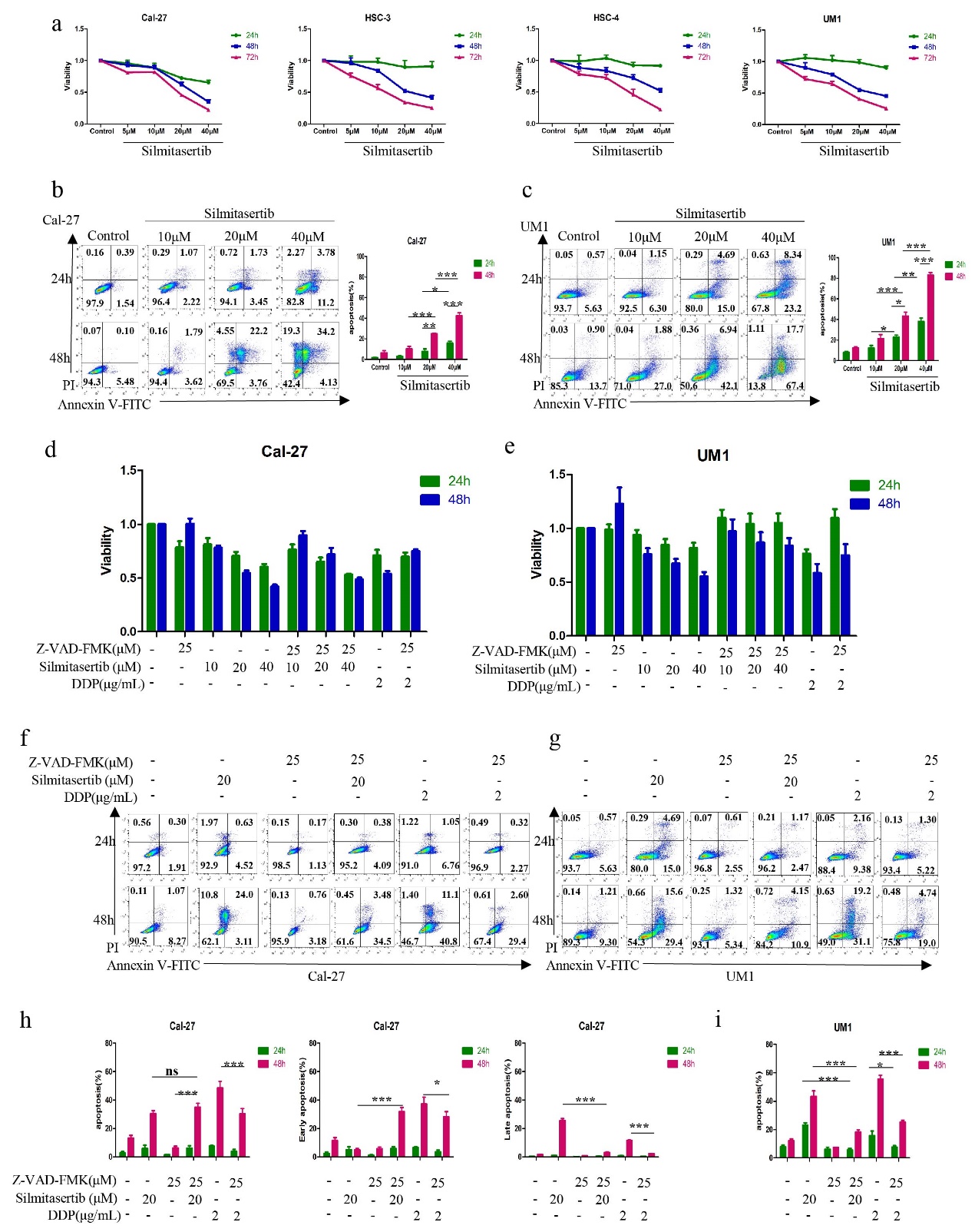
**

**Fig. S4** Silmitasertib can promote OSCC cell apoptosis. **(a)** Cell viability of four OSCC cell lines treated with 5, 10, 20, 40 μM silmitasertib for 24, 48, 72 h were measured by CCK8 assay. **(b, c)** Apoptosis in Cal-27 and UM1 cells treated with 10, 20, 40 μM silmitasertib for 24, 48h was conducted using FITC/PI double staining. And rates of cell apoptosis were measured. **(d, e)** Cell viability of Cal-27 and UM1 cells treated with 25 μM Z-VAD-FMK (apoptosis inhibitor), 10/20/40 μM silmitasertib or 25 μM Z-VAD-FMK +10/20/40 μM silmitasertib for 24, 48 h were measured by CCK8 assay. The 2 μg/ml DDP group was positive control. **(f, g)** Apoptosis in Cal-27 and UM1 cells treated with 25 μM Z-VAD-FMK, 20 μM silmitasertib or 25 μM Z-VAD-FMK +20 μM silmitasertib for 24, 48h was conducted using FITC/PI double staining. The 2 μg/ml DDP group was positive control. **(h, i)** Rates of cell apoptosis (including early/late apoptosis) were measured in different groups in Cal-27 and UM1 cells. Data are shown as mean ± SD {(*) P < 0.05, (**) P < 0.01, (***) P < 0.001} from three replicates.

We explored the toxic effect of silmitasertib on OSCC cells. First, we tested whether silmitasertib has an inhibition effect on four OSCC cells (HSC-3, HSC-4, Cal-27, UM1). The concentration gradient of silmitasertib was set to 0, 5, 10, 20, 40 µM, and CCK8 was used to test cell viability at 24, 48, and 72 h, respectively. The cell viability of the four OSCC cell lines (HSC-3, HSC-4, Cal-27, UM1) all show a time-dependent and concentration-dependent decline **(Fig. S4a)**. Next, we selected Cal-27 and UM1 cells to verify whether silmitasertib can increase apoptosis. We treated the cells with 10, 20, and 40 µM silmitasertib, and double-stained the cells with FITC/PI at 24 and 48 h, respectively, and then detected the proportion of apoptotic cells by flow cytometry. In Cal-27, the apoptosis rate of cells treated with the same concentration of silmitasertib gradually increased from 24h to 48h, and the apoptosis rate caused by 20,40μM silmitasertib was statistically different at 24 and 48h, respectively, P< 0.05 and P<0.001**(Fig. S4b)**. At the same time point, as the concentration of silmitasertib increased, the rate of apoptosis gradually increased. At 24 h, 20 and 40μM silmitasertib caused a statistically different rate of apoptosis, P<0.05**(Fig.S4b)**. And at 48h, 10 and 20, 20 and 40μM silmitasertib induced a statistically significant difference in the rate of apoptosis, both P<0.001 **(Fig. S4b)**. The change trend of apoptosis rate of UM1 cells treated with different concentrations of silentasertib at different time points is basically the same as that of Cal-27 cells **(Fig. S4c)**. Therefore, we can preliminarily determine that silmitasertib can promote the apoptosis of OSCC cells with time and concentration dependence.

To further confirm the results we obtained, we pretreated Cal-27 and UM1 cells with 25μM apoptosis inhibitor Z-VAD-FMK for 2 h, then treated the cells with silmitasertib, and then used the CCK8 test to detect changes in cell viability. Although the pretreatment of Z-VAD-FMK did not obviously restore the cell viability of Cal-27, it obviously restored the cell viability of UM1**(Fig. S4d, S4e)**. Next, we pretreated the above two cells with 25μM Z-VAD-FMK, then treated the cells with 20μM silmitasertib, and measured the proportion of apoptosis by flow cytometry at 24 and 48 h. Although the pretreatment of Z-VAD-FMK did not reduce the total apoptosis rate of Cal-27, most of the apoptotic cells in the silmitasertib+Z-VAD-FMK group are in an early apoptotic state, while they were in the late apoptotic state in the silmitasertib group at 48h. This indicates that Z-VAD-FMK may inhibit the progression of apoptosis **(Fig. S4f, S4h)**. In addition, at 24 and 48 h, Z-VAD-FMK pretreatment reduced the total apoptosis rate of UM1, and it was more significant at 48 h **(Fig. S4g, S4i)**. These results indicate that silmitasertib could induce or partially induce the caspase dependent apoptosis.
